# 2,2,2-Tribromo-*N*-(2-methyl­phen­yl)acetamide

**DOI:** 10.1107/S1600536810009852

**Published:** 2010-03-20

**Authors:** B. Thimme Gowda, Sabine Foro, P. A. Suchetan, Hartmut Fuess

**Affiliations:** a Department of Chemistry, Mangalore University, Mangalagangotri 574 199, Mangalore, India; b Institute of Materials Science, Darmstadt University of Technology, Petersenstrasse 23, D-64287 Darmstadt, Germany

## Abstract

The asymmetric unit of the title compound, C_9_H_8_Br_3_NO, contains two independent mol­ecules. Intra­molecular N—H⋯Br hydrogen bonds are present in both mol­ecules. In the crystal, mol­ecules are packed into columnar chains by inter­molecular N—H⋯O hydrogen bonds.

## Related literature

For preparation of the title compound, see: Gowda *et al.* (2003 ▶). For background to our study of the effect of ring and side-chain substituents on the solid state structures of *N*-aromatic amides and for related structures, see: Brown (1966 ▶); Gowda *et al.* (2009 ▶, 2010 ▶).
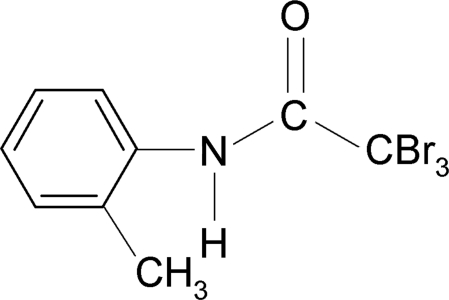


## Experimental

### 

#### Crystal data

C_9_H_8_Br_3_NO
*M* *_r_* = 385.89
Monoclinic, 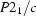

*a* = 9.949 (2) Å
*b* = 21.429 (4) Å
*c* = 11.653 (2) Å
β = 107.69 (1)°
*V* = 2366.9 (8) Å^3^
*Z* = 8
Cu *K*α radiation
μ = 12.40 mm^−1^
*T* = 299 K
0.55 × 0.28 × 0.28 mm

#### Data collection

Enraf–Nonius CAD-4 diffractometer
Absorption correction: ψ scan (North *et al.*, 1968 ▶) *T*_min_ = 0.056, *T*_max_ = 0.129
5721 measured reflections
4204 independent reflections
3666 reflections with *I* > 2σ(*I*)
*R*_int_ = 0.149
3 standard reflections every 120 min  intensity decay: 1.5%

#### Refinement

*R*[*F* ^2^ > 2σ(*F* ^2^)] = 0.073
*wR*(*F* ^2^) = 0.212
*S* = 1.05
4204 reflections
260 parameters
2 restraints
H atoms treated by a mixture of independent and constrained refinement
Δρ_max_ = 1.77 e Å^−3^
Δρ_min_ = −1.34 e Å^−3^

### 

Data collection: *CAD-4-PC* (Enraf–Nonius, 1996 ▶); cell refinement: *CAD-4-PC*; data reduction: *REDU4* (Stoe & Cie, 1987 ▶); program(s) used to solve structure: *SHELXS97* (Sheldrick, 2008 ▶); program(s) used to refine structure: *SHELXL97* (Sheldrick, 2008 ▶); molecular graphics: *PLATON* (Spek, 2009 ▶); software used to prepare material for publication: *SHELXL97*.

## Supplementary Material

Crystal structure: contains datablocks I, global. DOI: 10.1107/S1600536810009852/pk2229sup1.cif

Structure factors: contains datablocks I. DOI: 10.1107/S1600536810009852/pk2229Isup2.hkl

Additional supplementary materials:  crystallographic information; 3D view; checkCIF report

## Figures and Tables

**Table 1:** Hydrogen-bond geometry (Å, °)

*D*—H⋯*A*	*D*—H	H⋯*A*	*D*⋯*A*	*D*—H⋯*A*
N1—H1*N*⋯O2	0.84 (4)	2.28 (6)	3.031 (8)	149 (9)
N1—H1*N*⋯Br1	0.84 (4)	2.53 (9)	3.048 (7)	120 (8)
N2—H2*N*⋯O1^i^	0.85 (4)	2.23 (7)	2.928 (9)	139 (9)
N2—H2*N*⋯Br5	0.85 (4)	2.50 (9)	3.036 (6)	122 (8)

Symmetry code: (i) 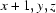.

**Fig. 1.**
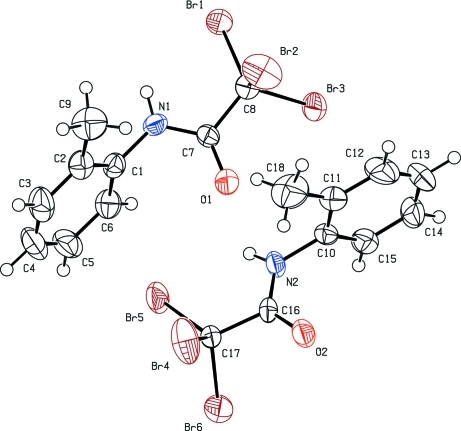
Molecular structure of the title compound, showing the atom labelling scheme. Displacement ellipsoids are drawn at the 50% probability level and H atoms are represented as small spheres of arbitrary radius.

**Fig. 2.**
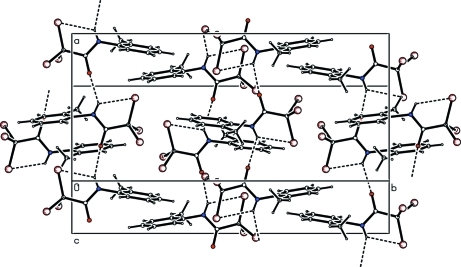
Molecular packing in the structure of the title compound with hydrogen bonds shown as dashed lines.

## supplementary crystallographic information

### Comment

As part of a study of the effect of ring and the side chain substituents on the solid state structures of *N*-aromatic amides (Gowda *et al.*, 2009, 2010), the structure of 2,2,2-tribromo-*N*-(2-methylphenyl)acetamide has been determined (Fig.1). The asymmetric unit of the structure contains two independent molecules. The conformation of the N—H bond in the structure is *anti* to the C=O bond in the side chain, similar to that observed in 2,2,2-tribromo-*N*-(phenyl)acetamide, 2,2,2-tribromo-*N*-(2-chlorophenyl)acetamide (Gowda *et al.*, 2010), 2,2,2-tribromo-*N*-(3-methylphenyl)acetamide (Gowda *et al.*, 2009) and other amides (Brown, 1966). The structure of the title compound shows both intramolecular N—H···Br and intermolecular N—H···O H-bonding. A packing diagram of molecules showing the hydrogen bonds N1—H1N···O2 and N2—H2N···O1 (Table 1) involved in the formation of molecular chains is shown in Fig. 2.

### Experimental

The title compound was prepared from *o*-toluidine, tribromoacetic acid and phosphorylchloride according to the literature method (Gowda *et al.*, 2003). The purity of the compound was checked by determining its melting point. It was further characterized by recording its infrared spectra. Single crystals of the title compound used for X-ray diffraction studies were obtained by a slow evaporation of its solution in petroleum ether at room temperature.

### Refinement

The H atoms of the NH groups were located in a difference map and later restrained to the distance N—H = 0.86 (4) Å. The other H atoms were positioned with idealized geometry using a riding model with C—H = 0.93–0.96 Å. All H atoms were refined with isotropic displacement parameters (set to 1.2 times of the U_eq_ of the parent atom).

The largest residual electron-density features are located in the region of Br5 and Br6. The highest peak is 0.84 Å from Br5 and the deepest hole is 0.82 Å from Br6.

### Crystal data

**Table:**

C_9_H_8_Br_3_NO	*F*(000) = 1456
*M**_r_* = 385.89	*D*_x_ = 2.166 Mg m^−^^3^
Monoclinic, *P*2_1_/*c*	Cu *K*α radiation, λ = 1.54180 Å
Hall symbol: -P 2ybc	Cell parameters from 25 reflections
*a* = 9.949 (2) Å	θ = 4.1–18.8°
*b* = 21.429 (4) Å	µ = 12.40 mm^−^^1^
*c* = 11.653 (2) Å	*T* = 299 K
β = 107.69 (1)°	Rod, colourless
*V* = 2366.9 (8) Å^3^	0.55 × 0.28 × 0.28 mm
*Z* = 8	

### Data collection

**Table:**

Enraf–Nonius CAD-4 diffractometer	3666 reflections with *I* > 2σ(*I*)
Radiation source: fine-focus sealed tube	*R*_int_ = 0.149
graphite	θ_max_ = 67.0°, θ_min_ = 4.1°
ω/2θ scans	*h* = −11→3
Absorption correction: ψ scan (North *et al.*, 1968)	*k* = −25→0
*T*_min_ = 0.056, *T*_max_ = 0.129	*l* = −13→13
5721 measured reflections	3 standard reflections every 120 min
4204 independent reflections	intensity decay: 1.5%

### Refinement

**Table:**

Refinement on *F*^2^	Secondary atom site location: difference Fourier map
Least-squares matrix: full	Hydrogen site location: inferred from neighbouring sites
*R*[*F*^2^ > 2σ(*F*^2^)] = 0.073	H atoms treated by a mixture of independent and constrained refinement
*wR*(*F*^2^) = 0.212	*w* = 1/[σ^2^(*F*_o_^2^) + (0.1206*P*)^2^ + 14.5272*P*] where *P* = (*F*_o_^2^ + 2*F*_c_^2^)/3
*S* = 1.05	(Δ/σ)_max_ = 0.001
4204 reflections	Δρ_max_ = 1.77 e Å^−^^3^
260 parameters	Δρ_min_ = −1.33 e Å^−^^3^
2 restraints	Extinction correction: *SHELXL97* (Sheldrick, 2008), Fc^*^=kFc[1+0.001xFc^2^λ^3^/sin(2θ)]^-1/4^
Primary atom site location: structure-invariant direct methods	Extinction coefficient: 0.00203 (19)

### Special details

**Table:**

Geometry. All esds (except the esd in the dihedral angle between two l.s. planes) are estimated using the full covariance matrix. The cell esds are taken into account individually in the estimation of esds in distances, angles and torsion angles; correlations between esds in cell parameters are only used when they are defined by crystal symmetry. An approximate (isotropic) treatment of cell esds is used for estimating esds involving l.s. planes.
Refinement. Refinement of *F*^2^ against ALL reflections. The weighted *R*-factor wR and goodness of fit *S* are based on *F*^2^, conventional *R*-factors *R* are based on *F*, with *F* set to zero for negative *F*^2^. The threshold expression of *F*^2^ > σ(*F*^2^) is used only for calculating *R*-factors(gt) etc. and is not relevant to the choice of reflections for refinement. *R*-factors based on *F*^2^ are statistically about twice as large as those based on *F*, and *R*- factors based on ALL data will be even larger.

### Fractional atomic coordinates and isotropic or equivalent isotropic displacement parameters (Å^2^)

**Table:**

	*x*	*y*	*z*	*U*_iso_*/*U*_eq_	
C1	−0.0153 (8)	0.3501 (4)	0.6789 (7)	0.0360 (16)	
C2	0.0276 (9)	0.3149 (4)	0.7841 (7)	0.0428 (18)	
C3	0.0010 (11)	0.2519 (5)	0.7730 (10)	0.059 (2)	
H3	0.0286	0.2272	0.8418	0.071*	
C4	−0.0656 (12)	0.2238 (5)	0.6631 (12)	0.063 (3)	
H4	−0.0811	0.1809	0.6587	0.076*	
C5	−0.1078 (11)	0.2593 (5)	0.5620 (11)	0.061 (3)	
H5	−0.1535	0.2410	0.4881	0.073*	
C6	−0.0822 (9)	0.3234 (4)	0.5694 (8)	0.0453 (19)	
H6	−0.1104	0.3480	0.5004	0.054*	
C7	−0.0894 (7)	0.4574 (3)	0.6711 (7)	0.0335 (15)	
C8	−0.0422 (8)	0.5264 (4)	0.6954 (6)	0.0356 (15)	
C9	0.0957 (12)	0.3450 (6)	0.9028 (9)	0.065 (3)	
H9A	0.0317	0.3747	0.9196	0.078*	
H9B	0.1800	0.3661	0.9006	0.078*	
H9C	0.1191	0.3138	0.9647	0.078*	
N1	0.0124 (6)	0.4153 (3)	0.6855 (6)	0.0369 (14)	
H1N	0.095 (5)	0.429 (4)	0.700 (9)	0.044*	
O1	−0.2153 (6)	0.4451 (3)	0.6442 (7)	0.0537 (16)	
Br1	0.14164 (9)	0.54417 (4)	0.68400 (10)	0.0519 (3)	
Br2	−0.04344 (14)	0.54241 (6)	0.85827 (9)	0.0703 (4)	
Br3	−0.17695 (10)	0.57952 (5)	0.58669 (10)	0.0581 (4)	
C10	0.5167 (7)	0.4983 (4)	0.7302 (7)	0.0350 (15)	
C11	0.5752 (9)	0.5236 (4)	0.8425 (8)	0.0464 (19)	
C12	0.5630 (12)	0.5873 (6)	0.8526 (11)	0.068 (3)	
H12	0.6000	0.6059	0.9277	0.082*	
C13	0.4978 (12)	0.6242 (5)	0.7546 (13)	0.069 (3)	
H13	0.4913	0.6671	0.7636	0.082*	
C14	0.4424 (10)	0.5969 (5)	0.6435 (11)	0.059 (3)	
H14	0.3986	0.6215	0.5769	0.070*	
C15	0.4512 (9)	0.5338 (4)	0.6306 (8)	0.0439 (18)	
H15	0.4136	0.5152	0.5555	0.053*	
C16	0.4364 (7)	0.3930 (3)	0.7382 (7)	0.0338 (15)	
C17	0.4640 (7)	0.3218 (4)	0.7325 (7)	0.0354 (16)	
C18	0.6471 (13)	0.4835 (7)	0.9492 (10)	0.075 (3)	
H18A	0.7243	0.4616	0.9341	0.090*	
H18B	0.5809	0.4539	0.9624	0.090*	
H18C	0.6822	0.5093	1.0194	0.090*	
N2	0.5275 (7)	0.4317 (3)	0.7144 (6)	0.0370 (14)	
H2N	0.591 (8)	0.416 (4)	0.689 (8)	0.044*	
O2	0.3318 (7)	0.4088 (3)	0.7643 (7)	0.0539 (16)	
Br4	0.48061 (16)	0.28822 (6)	0.88797 (10)	0.0740 (4)	
Br5	0.63133 (11)	0.30070 (5)	0.68942 (12)	0.0628 (4)	
Br6	0.30461 (11)	0.28588 (5)	0.61417 (12)	0.0677 (4)	

### Atomic displacement parameters (Å^2^)

**Table:**

	*U*^11^	*U*^22^	*U*^33^	*U*^12^	*U*^13^	*U*^23^
C1	0.033 (3)	0.036 (4)	0.042 (4)	0.005 (3)	0.016 (3)	0.006 (3)
C2	0.046 (4)	0.045 (5)	0.044 (4)	0.005 (4)	0.024 (3)	0.004 (3)
C3	0.070 (6)	0.045 (5)	0.072 (7)	0.013 (5)	0.034 (5)	0.019 (5)
C4	0.079 (7)	0.028 (5)	0.096 (8)	−0.001 (4)	0.046 (6)	−0.004 (5)
C5	0.064 (6)	0.043 (5)	0.079 (7)	−0.008 (4)	0.029 (5)	−0.022 (5)
C6	0.049 (4)	0.046 (5)	0.040 (4)	0.006 (4)	0.013 (3)	0.004 (3)
C7	0.035 (4)	0.027 (4)	0.041 (4)	−0.002 (3)	0.015 (3)	0.007 (3)
C8	0.038 (4)	0.040 (4)	0.032 (3)	0.001 (3)	0.015 (3)	0.000 (3)
C9	0.069 (6)	0.078 (8)	0.043 (5)	−0.004 (6)	0.010 (4)	0.010 (5)
N1	0.026 (3)	0.039 (4)	0.044 (4)	−0.002 (3)	0.008 (2)	0.008 (3)
O1	0.038 (3)	0.036 (3)	0.087 (5)	0.002 (2)	0.019 (3)	0.004 (3)
Br1	0.0403 (5)	0.0394 (6)	0.0790 (7)	−0.0034 (3)	0.0227 (4)	0.0118 (4)
Br2	0.1006 (9)	0.0767 (8)	0.0412 (6)	−0.0170 (6)	0.0330 (5)	−0.0102 (5)
Br3	0.0506 (5)	0.0381 (6)	0.0706 (7)	0.0069 (4)	−0.0041 (4)	0.0126 (4)
C10	0.032 (3)	0.030 (4)	0.045 (4)	−0.005 (3)	0.015 (3)	0.000 (3)
C11	0.041 (4)	0.045 (5)	0.052 (5)	−0.006 (4)	0.011 (3)	−0.005 (4)
C12	0.070 (6)	0.060 (7)	0.071 (7)	−0.017 (5)	0.014 (5)	−0.035 (5)
C13	0.068 (6)	0.029 (5)	0.107 (9)	−0.008 (4)	0.025 (6)	−0.006 (5)
C14	0.055 (5)	0.037 (5)	0.085 (7)	0.008 (4)	0.023 (5)	0.019 (5)
C15	0.044 (4)	0.040 (5)	0.047 (4)	−0.004 (3)	0.015 (3)	0.001 (3)
C16	0.033 (3)	0.027 (4)	0.042 (4)	0.009 (3)	0.012 (3)	0.001 (3)
C17	0.030 (3)	0.027 (4)	0.049 (4)	0.002 (3)	0.012 (3)	0.003 (3)
C18	0.074 (7)	0.090 (9)	0.046 (5)	−0.002 (6)	−0.003 (5)	0.006 (5)
N2	0.041 (3)	0.023 (3)	0.053 (4)	0.005 (3)	0.023 (3)	0.001 (3)
O2	0.049 (3)	0.031 (3)	0.091 (5)	0.003 (3)	0.036 (3)	0.000 (3)
Br4	0.1248 (11)	0.0556 (7)	0.0497 (6)	0.0301 (6)	0.0387 (6)	0.0187 (5)
Br5	0.0549 (6)	0.0364 (6)	0.1123 (9)	0.0086 (4)	0.0482 (6)	−0.0001 (5)
Br6	0.0544 (6)	0.0506 (7)	0.0818 (8)	0.0001 (4)	−0.0036 (5)	−0.0176 (5)

### Geometric parameters (Å, °)

**Table:**

C1—C6	1.371 (12)	C10—C11	1.372 (12)
C1—C2	1.391 (11)	C10—C15	1.375 (12)
C1—N1	1.423 (11)	C10—N2	1.446 (10)
C2—C3	1.374 (14)	C11—C12	1.377 (15)
C2—C9	1.490 (14)	C11—C18	1.503 (14)
C3—C4	1.389 (17)	C12—C13	1.378 (18)
C3—H3	0.9300	C12—H12	0.9300
C4—C5	1.358 (17)	C13—C14	1.374 (17)
C4—H4	0.9300	C13—H13	0.9300
C5—C6	1.396 (14)	C14—C15	1.366 (13)
C5—H5	0.9300	C14—H14	0.9300
C6—H6	0.9300	C15—H15	0.9300
C7—O1	1.224 (9)	C16—O2	1.217 (9)
C7—N1	1.329 (10)	C16—N2	1.320 (10)
C7—C8	1.552 (11)	C16—C17	1.554 (10)
C8—Br1	1.912 (7)	C17—Br4	1.910 (8)
C8—Br3	1.914 (8)	C17—Br6	1.918 (8)
C8—Br2	1.932 (7)	C17—Br5	1.933 (7)
C9—H9A	0.9600	C18—H18A	0.9600
C9—H9B	0.9600	C18—H18B	0.9600
C9—H9C	0.9600	C18—H18C	0.9600
N1—H1N	0.84 (4)	N2—H2N	0.85 (4)
			
C6—C1—C2	121.8 (8)	C11—C10—C15	122.7 (8)
C6—C1—N1	119.4 (7)	C11—C10—N2	119.0 (7)
C2—C1—N1	118.8 (7)	C15—C10—N2	118.3 (7)
C3—C2—C1	116.8 (8)	C10—C11—C12	116.8 (9)
C3—C2—C9	122.2 (9)	C10—C11—C18	121.3 (9)
C1—C2—C9	121.0 (9)	C12—C11—C18	121.9 (10)
C2—C3—C4	122.5 (9)	C11—C12—C13	121.9 (10)
C2—C3—H3	118.7	C11—C12—H12	119.0
C4—C3—H3	118.7	C13—C12—H12	119.0
C5—C4—C3	119.5 (9)	C14—C13—C12	119.3 (9)
C5—C4—H4	120.3	C14—C13—H13	120.4
C3—C4—H4	120.3	C12—C13—H13	120.4
C4—C5—C6	119.7 (10)	C15—C14—C13	120.3 (10)
C4—C5—H5	120.1	C15—C14—H14	119.9
C6—C5—H5	120.1	C13—C14—H14	119.9
C1—C6—C5	119.7 (9)	C14—C15—C10	119.0 (9)
C1—C6—H6	120.2	C14—C15—H15	120.5
C5—C6—H6	120.2	C10—C15—H15	120.5
O1—C7—N1	124.6 (7)	O2—C16—N2	124.8 (7)
O1—C7—C8	118.8 (7)	O2—C16—C17	117.4 (7)
N1—C7—C8	116.5 (6)	N2—C16—C17	117.8 (6)
C7—C8—Br1	114.8 (5)	C16—C17—Br4	107.1 (5)
C7—C8—Br3	109.5 (5)	C16—C17—Br6	107.8 (5)
Br1—C8—Br3	109.2 (4)	Br4—C17—Br6	110.2 (4)
C7—C8—Br2	104.8 (5)	C16—C17—Br5	114.7 (5)
Br1—C8—Br2	109.0 (4)	Br4—C17—Br5	109.0 (4)
Br3—C8—Br2	109.4 (4)	Br6—C17—Br5	108.0 (4)
C2—C9—H9A	109.5	C11—C18—H18A	109.5
C2—C9—H9B	109.5	C11—C18—H18B	109.5
H9A—C9—H9B	109.5	H18A—C18—H18B	109.5
C2—C9—H9C	109.5	C11—C18—H18C	109.5
H9A—C9—H9C	109.5	H18A—C18—H18C	109.5
H9B—C9—H9C	109.5	H18B—C18—H18C	109.5
C7—N1—C1	122.2 (6)	C16—N2—C10	120.8 (6)
C7—N1—H1N	117 (7)	C16—N2—H2N	117 (7)
C1—N1—H1N	120 (7)	C10—N2—H2N	122 (7)
			
C6—C1—C2—C3	0.6 (12)	C15—C10—C11—C12	1.4 (12)
N1—C1—C2—C3	−178.8 (7)	N2—C10—C11—C12	179.5 (8)
C6—C1—C2—C9	−177.9 (8)	C15—C10—C11—C18	−179.4 (9)
N1—C1—C2—C9	2.7 (11)	N2—C10—C11—C18	−1.3 (12)
C1—C2—C3—C4	−0.1 (14)	C10—C11—C12—C13	−1.2 (15)
C9—C2—C3—C4	178.4 (9)	C18—C11—C12—C13	179.7 (11)
C2—C3—C4—C5	−0.6 (16)	C11—C12—C13—C14	0.3 (17)
C3—C4—C5—C6	0.8 (15)	C12—C13—C14—C15	0.4 (16)
C2—C1—C6—C5	−0.4 (12)	C13—C14—C15—C10	−0.2 (14)
N1—C1—C6—C5	179.0 (8)	C11—C10—C15—C14	−0.7 (12)
C4—C5—C6—C1	−0.3 (14)	N2—C10—C15—C14	−178.9 (7)
O1—C7—C8—Br1	−160.3 (6)	O2—C16—C17—Br4	−59.3 (8)
N1—C7—C8—Br1	21.9 (8)	N2—C16—C17—Br4	120.7 (6)
O1—C7—C8—Br3	−37.2 (9)	O2—C16—C17—Br6	59.3 (8)
N1—C7—C8—Br3	145.1 (6)	N2—C16—C17—Br6	−120.7 (6)
O1—C7—C8—Br2	80.1 (8)	O2—C16—C17—Br5	179.6 (6)
N1—C7—C8—Br2	−97.6 (6)	N2—C16—C17—Br5	−0.3 (9)
O1—C7—N1—C1	−4.5 (12)	O2—C16—N2—C10	6.5 (12)
C8—C7—N1—C1	173.0 (6)	C17—C16—N2—C10	−173.5 (7)
C6—C1—N1—C7	71.7 (10)	C11—C10—N2—C16	83.9 (9)
C2—C1—N1—C7	−108.9 (8)	C15—C10—N2—C16	−97.9 (9)

### Hydrogen-bond geometry (Å, °)

**Table:**

*D*—H···*A*	*D*—H	H···*A*	*D*···*A*	*D*—H···*A*
N1—H1N···O2	0.84 (4)	2.28 (6)	3.031 (8)	149 (9)
N1—H1N···Br1	0.84 (4)	2.53 (9)	3.048 (7)	120 (8)
N2—H2N···O1^i^	0.85 (4)	2.23 (7)	2.928 (9)	139 (9)
N2—H2N···Br5	0.85 (4)	2.50 (9)	3.036 (6)	122 (8)

Symmetry codes: (i) *x*+1, *y*, *z*.
